# Paired protein kinases PRKCI-RIPK2 promote pancreatic cancer growth and metastasis via enhancing NF-κB/JNK/ERK phosphorylation

**DOI:** 10.1186/s10020-023-00648-z

**Published:** 2023-04-04

**Authors:** Juying Jiao, Linjie Ruan, Chien-shan Cheng, Fengjiao Wang, Peiwen Yang, Zhen Chen

**Affiliations:** 1 Department of Integrative Oncology, Fudan University Shanghai Cancer Center, No. 270 Dongan Rd., Xuhui District, Shanghai, 200032 China; 2 Department of Oncology, Shanghai Medical College, Fudan University, Shanghai, 200032 China

**Keywords:** Pancreatic cancer, Protein kinase, Receptor interacting protein kinase 2, Protein kinase C iota, Phosphorylation

## Abstract

**Background:**

Protein kinases play a pivotal role in the malignant evolution of pancreatic cancer (PC) through mediating phosphorylation. Many kinase inhibitors have been developed and translated into clinical use, while the complex pathology of PC confounds their clinical efficacy and warrants the discovery of more effective therapeutic targets.

**Methods:**

Here, we used the Gene Expression Omnibus (GEO) database and protein kinase datasets to map the PC-related protein kinase-encoding genes. Then, applying Gene Expression and Profiling Interactive Analysis (GEPIA), GEO and Human Protein Atlas, we evaluated gene correlation, gene expression at protein and mRNA levels, as well as survival significance. In addition, we performed protein kinase RIPK2 knockout and overexpression to observe effects of its expression on PC cell proliferation, migration and invasion in vitro, as well as cell apoptosis, reactive oxygen species (ROS) production and autophagy. We established PC subcutaneous xenograft and liver metastasis models to investigate the effects of RIPK2 knockout on PC growth and metastasis. Co-immunoprecipitation and immunofluorescence were utilized to explore the interaction between protein kinases RIPK2 and PRKCI. Polymerase chain reaction and immunoblotting were used to evaluate gene expression and protein phosphorylation level.

**Results:**

We found fourteen kinases aberrantly expressed in human PC and nine kinases with prognosis significance. Among them, RIPK2 with both serine/threonine and tyrosine activities were validated to promote PC cells proliferation, migration and invasion. RIPK2 knockout could inhibit subcutaneous tumor growth and liver metastasis of PC. In addition, RIPK2 knockout suppressed autophagosome formation, increased ROS production and PC cell apoptosis. Importantly, another oncogenic kinase PRKCI could interact with RIPK2 to enhance the phosphorylation of downstream NF-κB, JNK and ERK.

**Conclusion:**

Paired protein kinases PRKCI-RIPK2 with multiple phosphorylation activities represent a new pathological mechanism in PC and could provide potential targets for PC therapy.

## Introduction

Bleak prognosis of pancreatic cancer (PC) have long been challenging the physicians and medical technologies. Multimodality strategies including surgery, chemotherapy, immunotherapy and targeted drugs are being developed to improve the quality of life and survival of this cancer patient population. Among them, molecular-driven drugs are expected to become a breakthrough point in PC control according to the latest expert group discussion (Milella et al. 2022). In fact, multi-dimensional molecular screening for diagnoses and treatments of PC springs up at an increasing rate.

Protein kinase was evidenced by emerging researches to drive tumor growth and desmoplasia in PC (Creeden et al. 2020). Tyrosine kinases such as epidermal growth factor receptor (EGFR) and mitogen activated protein kinase kinase (MAPKK, also known as MEK) have been intensively investigated and developed into small molecule inhibitors to use in clinics (Lakkakula et al. 2019). Serine/threonine kinases function in the middle and downstream of the signaling pathway to phosphorylate serine and/or threonine residues of their substrates, and thus initiate ensuing signal transduction. Protein kinase C (PKC), phosphoinositide 3 kinase (PI3K) and mitogen-activated protein kinase (MAPK), three representative star proteins with serine/threonine kinase activity, have long been the hotspots in tumor research and are the very concerned targets for PC therapy (Drosten and Barbacid 2020; Hobbs et al. 2020; Lin et al. 2021). Such kinases are often shared by receptor-mediated signaling with much cross-talk, and serve as the core functional machinery in tumor cells. Although many progresses have been made in this field, heterogeneity of tumor molecular profiles, side effects of key targets interference, cellular compensatory adaptation and other undetermined factors impede clinical transformation of many drugs directly targeting kinase activity regulation. A panoramic understanding and more nuanced mechanisms exploration on the role of protein kinases in PC will raise hopes in novel therapeutic strategies development.

The present study exploited the publicly accessible data from clinical samples to reveal the aberrantly expressed protein kinases profiles in PC. A pair of serine/threonine kinases PRKCI-RIPK2 were spotted to interactively regulate PC malignant behaviors. Further in-depth studies verified that PRKCI  can interact with RIPK2 to promote PC growth and liver metastasis through enhancing phosphorylation of JNK, ERK and NF-κB signaling pathways. The broad phosphorylation activity of PRKCI and RIPK2 in PC pathogenesis provides candidate targets for effective intervention.

## Materials and methods

### Differentially expressed protein kinase-encoding genes between PC and normal tissues

Datasets including PC samples and healthy control samples were retrieved from the Gene Expression Omnibus (GEO, https://www.ncbi.nlm.nih.gov/geo) database. GSE46234 and GSE 62165 datasets were used to screen the respectively differentially expressed genes (DEGs) between PC and normal tissues. The intersection of the above two datasets were set as the DEGs in this study. After that, a total of 518 identified protein kinases (Cowan-Jacob et al. 2006) were referred to determine the differential expressed protein kinase-encoding genes between PC and normal tissues.

### Enrichment, protein–protein interaction (PPI) and correlation analyses

Enrichment analysis based on the DEGs were performed by Gene Ontology (GO) and Kyoto Encyclopedia of Genes and Genomes (KEGG). The GO enrichment includes the main involved cellular component, biological process and molecular function. The KEGG enrichment indicates the main involved signaling pathways of the DEGs. The DEGs were investigated through the STRING database (https://cn.string-db.org) to construct a physical PPI network with setting the highest confidence of 0.9, and Cytoscape (v3.9.0) was used for visualization. The MCODE algorithm in Cytoscape was applied for modular analysis. Spearman correlation analyses of the expression of different genes were performed through the Gene Expression and Profiling Interactive Analysis (GEPIA, http://gepia.cancer-pku.cn/index.html) database.

### Gene expression and survival analysis

The differentially expressed protein kinase-encoding genes were subjected to GEPIA to compare their expression between PC and normal pancreatic tissues, and analyze their impact on the overall survival of PC patients. Protein expression by immunohistochemistry was obtained from the Human Protein Atlas (https://www.proteinatlas.org) database.

### Cell culture and drugs

Cell lines Panc1 and Mia paca2 were obtained from the American Type Culture Collection, and Panc 02 was obtained from Fudan University Shanghai Cancer Center. Cells were cultured in DMEM medium supplemented with 10% fetal bovine serum (Biological Industries, Israel), 100 μg/mL penicillin and streptomycin (Gibco, USA). Mia paca2 was additionally supplemented 5% horse serum in medium. Cells were maintained in a sterilized and humidified incubator at 37 ℃ with 5% CO_2_. PKC-iota inhibitor 1 (Topscience, Shanghai, China) was added in a concentration of 10 μM for Panc1 and 100 μM for Mia paca2. Chloroquine (Topscience, Shanghai, China) was used in a concentration of 10 μM. 3-Methyladenine (Topscience, Shanghai, China) was used in a concentration of 10 mM.

### Plasmid transfection for RIPK2 knockout and overexpression

A lentiviral vector mediated cell transfection method was used for stable RIPK2 (Gene ID: human, NM_003821.6; mouse, NM_138952) expression regulation. Panc1 and Mia paca2 were seeded into 6-well plates to allow them to proliferate into 2 × 10^5^ cells in each well for lentivirus transfection. For RIPK2 overexpression (RIPK2^OE^), lentiviral vector PGMLV-CMV-MCS-EF1-ZsGreen1-T2A-Puro vector (PGMLV-6395, Genomeditech, Shanghai, China) and control vectors (RIPK2^Mock^) were added with polybrene into cells with an MOI of 10 for Panc1 and 20 for Mia paca2 in 1 mL medium. For RIPK2 knockout (RIPK2^KO^), HBLV-h-RIPK2-cas9-gRNA-Puro vector (Hanbio, Shanghai, China) and control vector (RIPK2^Scramble^) were added with polybrene into cells with a MOI of 20 for Panc1 and Mia paca2 in 1 mL medium. After 24 h, medium containing lentivirus were changed with 3 mL fresh medium in each well to allow cell growth for another 2 days. Next, Medium containing 4 μg/mL puromycin was used to culture cells for 7 days to screen out the successfully transfected cells, followed by medium containing 2 μg/mL puromycin to maintain culture. The expressions of RIPK2 were validated by polymerase chain reaction and immunoblotting. Because of the poor tumorigenicity of Panc1 and Mia paca2 in nude mice after RIPK2 knockout, we constructed Panc 02 cells with RIPK2 knockout for animal experiments. A lentivirus mediated luciferase plasmid (Genechem, Shanghai, China) transfection was performed as the above method for live imaging. In addition, to avoid the fluorescence crosstalk between PGMLV-6395 plasmid and the detection reagents, we transiently transfected the pcDNA3.1(+)-H_RIPK2 plasmid (Genomeditech, Shanghai, China) with HG-TransGene™ transfection reagent for RIPK2 overexpression in apoptosis and reactive oxygen species (ROS) assays.

### Cell viability and colony formation assay

For cell viability assay, five thousand cells were seeded in 96-well plates with 100 μL medium in each well. CCK8 reagents (ShareBio, Shanghai, China) were added at 6, 12, 24, 36, 48, 60, 72 h after cell attachment to react for 1 h, and then the absorbance were measured at 450 nm wavelength by a multi-well plate reader (BioTek, Synergy H1, USA). For colony formation assay, five hundred cells were seeded in 6-well plates with 2 mL medium in each well; the culture medium was replaced every two days. After ten days cultivation, the medium was removed and cells were fixed with 4% neutral paraformaldehyde for 30 min, and then stained with crystal violet for 20 min. Colonies more than fifty cells were counted under a microscope (Olympus, Japan).

### Wound healing assay and transwell invasion assay

Wound healing assay was performed to observe the migration capability of cells. Cells were first seeded in a 6-well plate with the complete medium to allow them to grow into monolayer confluence, and then incubated in serum-free medium for 6 h starvation. The confluent monolayer cells were next scratched with a 10 μL pipet tip to generate two perpendicular wound lines, and photographed at 0 h and 36 h to observe cell mobility. Transwell chamber (8 μm pores, Corning, USA) was used to observe the invasion capability of cells. Cells were first cultured in serum-free medium overnight, then 5 × 10^4^ cells in 100 μL serum-free medium were seeded in the top chamber coated with matrigel. The bottom chamber of each well was added 800 μL complete medium, and cells were allowed to invade for 48 h. Subsequently, the chamber membranes with cells in the upper surface removed and the lower surface preserved were fixed with 4% paraformaldehyde for half an hour. Crystal violet was used to stain cells, and images were captured with a light microscope.

### Cell apoptosis and ROS level assays

Cells were stained with the 488-Annexin V and PI Apoptosis Kit (ShareBio, Shanghai, China) and tested by a flow cytometer (Becton, Dickinson and Company, USA) to analyze cell apoptosis rate. The Fluorescent probe DCFH-DA (Beyotime, Shanghai, China) was loaded into cells to evaluate ROS level by detecting DCF with a flow cytometer.

### PC xenograft and liver metastasis mouse models

Twenty-four 4–6 weeks old male C57BL/6 mice were obtained from and housed in the department of laboratory science of Fudan University. All animal experiments conform to the guidelines for care and use of experimental animals, and the experimental protocol was approved by the ethics committee of laboratory animals of Fudan University (2019JS-020). Mice were randomly divided into two groups (n = 6 in each group) and were respectively inoculated with RIPK2^Scramble^ and RIPK2^KO^ Panc 02 cells. For the subcutaneous tumor model, PC cells were suspended in serum-free medium in a density of 1 × 10^7^/mL, each mouse was subcutaneously inoculated with 0.2 mL cells suspension at the right shoulder blade. Mice tumor size was measured every three days. After 4 weeks, mice were sacrificed, and tumors were removed and retained for later use. For the liver metastasis model, mice were first anesthetized and a 0.5 cm incision was made in the splenic region. Each mouse was injected with 2 × 10^6^ cells suspended in 0.1 mL PBS into the spleen. Tumor metastases were observed every week through live imaging by injecting d-Luciferin potassium salt and observing with IVIS® Spectrum CT (PerkinElmer, USA). After 5 weeks, the intact liver tissues were removed and observed at the end of experiments.

### Immunoblotting and quantitative polymerase chain reaction (qPCR)

Protein samples were prepared by RIPA lysis (Beyotime, Shanghai, China) added with proteinase and phosphatase inhibitor cocktail (TargetMol, Shanghai, China). Protein concentration were determined by BCA protein quantitation kit (Beyotime, Shanghai, China). Equal amount of proteins were subjected to polyacrylamide gel electrophoresis, and transferred to polyvinylidene fluoride membranes. The membranes were blocked in fast protein-blocking solution and then incubated with the following primary antibodies: RIPK2 (1:1000, CST), RIPK2 (1:500, Affinity), PRKCI (1:1000, Proteintech), LC3A/B (1:1000, CST), p62 (1:1000, CST), β-actin (1:10000, Affinity), NF-κB p65 (1:1000, CST), phosphor-NF-κB p65 (Ser276, 1:1000, Abways technology; Ser536, 1:1000, abcam), p44/42 MAPK(Erk1/2, 1:1000, CST), SAPK/JNK (1:1000, CST), p38 MAPK (1:1000, CST), phospho-p38 MAPK (Thr180/Thr182, 1:1000, CST), phosphor-p44/42 MAPK (Erk1/2, Thr202/Tyr204, 1:2000), phosphor-SAPK/JNK (Thr183/Tyr185, 1:1000, CST). After that, HRP-linked secondary antibody (1:1000, CST) and Immobilon Western HRP substrates (Millipore, Germany) were used for chemiluminescence under the gel imaging system (Tanon, Shanghai, China). qPCR was conducted with the commercially available RNA extraction, transcription and SYBR Green® Premix Pro Taq HS qPCR kits (Accurate biology, China). Programs for reaction were as follows: 95 ℃, 30 s for 1 cycle; 95 ℃, 5 s and 60 ℃, 30 s for 40 cycles. The following Primers were used, RIPK1-F: GGGAAGGTGTCTCTGTGTTTC, RIPK1-R: CCTCGTTGTGCTCAATGCAG; RIPK2-F: ATCCCGTACCACAAGCTCG, RIPK2-R: GGATGTGTAGGTGCTTCACTG; RIPK3-F: CATAGGAAGTGGGGCTACGAT, RIPK3-R: AATTCGTTATCCAGACTTGCCAT; RIPK4-F: GATCTCCGGTTCCGAATCATC, RIPK4-R: TCAGAAATCTTGACGTGGTAGTG; DSTYK-F: GACTGCCTCCCTTGCATACTG, DSTYK-R: CGAGTCTGAGTCCCATAGGTGA; LRRK1-F: TGGAGATGGTCCGCTACCTAC, LRRK1-R: TGTGTCCAAAATACGCTGCCA; LRRK2-F: ATGAGTGGCAATGTCAGGTGT, LRRK2-R: AATGTAAGCCTATGGAGCAAACA; ACTB-F: CATGTACGTTGCTATCCAGGC, ACTB-R: CTCCTTAATGTCACGCACGAT.

### Co-immunoprecipitation

Protein samples were prepared as methods in immunoblotting. Antibodies for bait protein were added in a concentration of 5 μg/mg and incubated overnight on the rotation mixer at 4 ℃. The Rabbit IgG isotype control (Abways technology, Shanghai, China) were incubated simultaneously as negative control. Then, Protein A/G Co-IP magnetic beads (ShareBio, Shanghai, China) were applied to capture the bait protein at 4 ℃ for 2 h, and the formed immunocomplex was separated with a magnetic frame. PBS supplemented with 1% TritonX-100 were used to wash the immunocomplex for 4 times, and finally, the immunocomplex was mixed with the loading buffer to heat for 5 min at 95 ℃ in a metal bath to elute the protein complex. The obtained proteins were subjected to immunoblotting as described above.

### Immunohistochemistry and immunofluorescence staining

For immunohistochemistry, tissues were embedded with paraffin and cut into 4 μm slides. Hydrogen peroxide was used to eliminate endogenous peroxidase activity, and citrate buffer solution was used to repair antigen under high-temperature and high-pressure conditions. Next, tissue slides were blocked with 5% goat serum and incubated with primary antibody overnight, followed by biotinylated secondary antibody incubation. Horseradish enzymes labeled streptomycin avidin solution and DAB chromogenic agents were used for visualization under a light microscope. For immunofluorescence observation, slides were prepared by cell crawling to approximately 80% confluence, then the 4% paraformaldehyde and 0.5% Triton X-100 were used for cell fixation and permeabilization respectively. After blocking with goat serum, slides were incubated with primary antibody in a humidified chamber overnight and followed by fluorescence labeled secondary antibody. Finally, the cell nucleus was counter-stained with DAPI, and the slides were sealed with anti-fluorescence quenching agent. Fluorescence images were observed in 30 min under a confocal microscope.

### Statistical analysis

The Student’s t-test was applied to compare differences between two independent groups, and one-way analysis of variance for more than two groups. A p-value of less than 0.05 was considered statistically different.

## Results

### DEGs between PC and normal tissues and enrichment analyses.

**Fig. 1:**
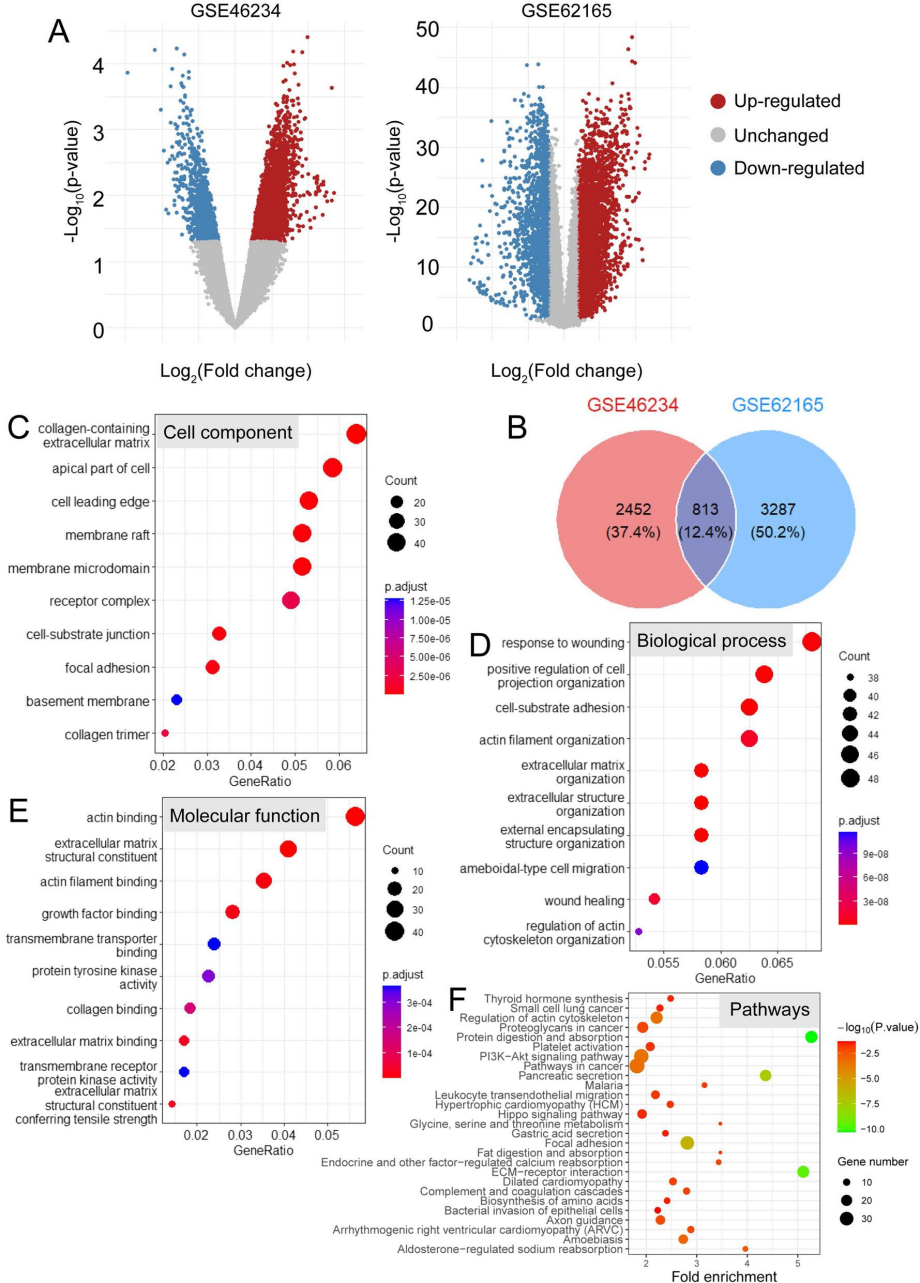
Differentially expressed genes (DEGs) screening between pancreatic cancer and normal tissues. **A** Volcano plot indicating respective DEGs of two datasets from the GEO database (|log2 fold change| ≥ 1, p < 0.05). **B** Venn diagram indicating the intersection of the two datasets. **C**–**E** Cell component, biological process and molecular function enriched by GO analysis based on the obtained common DEGs. **F** KEGG enrichment analysis based on the obtained common DEGs

There were 3265 and 4100 DEGs between PC and normal tissues in GSE46236 and GSE62165 respectively (|log2 fold change| ≥ 1, p < 0.05, Fig. 1A, B), in which totally 813 DEGs were shared by the both datasets (Fig. 1B). GO analyses indicate that the 813 DEGs were enriched in collagen-containing extracellular matrix, receptor complex, focal adhesion, etc. by cell component (p < 0.01, Fig. 1C); response to wounding, positive regulation of cell organization, cell-substrate adhesion, etc. by biological process (p < 0.01, Fig. 1D); transmembrane transporter binding, protein tyrosine kinase activity, transmembrane receptor protein kinase activity, etc. by molecular function (p < 0.01, Fig. 1E). KEGG analysis indicates these DEGs were mainly involved in pathways in cancer, PI3K-Akt signaling pathway, proteoglycans in cancer, focal adhesion, ECM-receptor interaction, glycine, serine and threonine metabolism, etc. (p < 0.05, Fig. 1F) These results suggest an intimate relationship between DEGs and malignant PC behavior.

### Hub genes in the DEGs and protein kinase-encoding genes screening

**Fig. 2:**
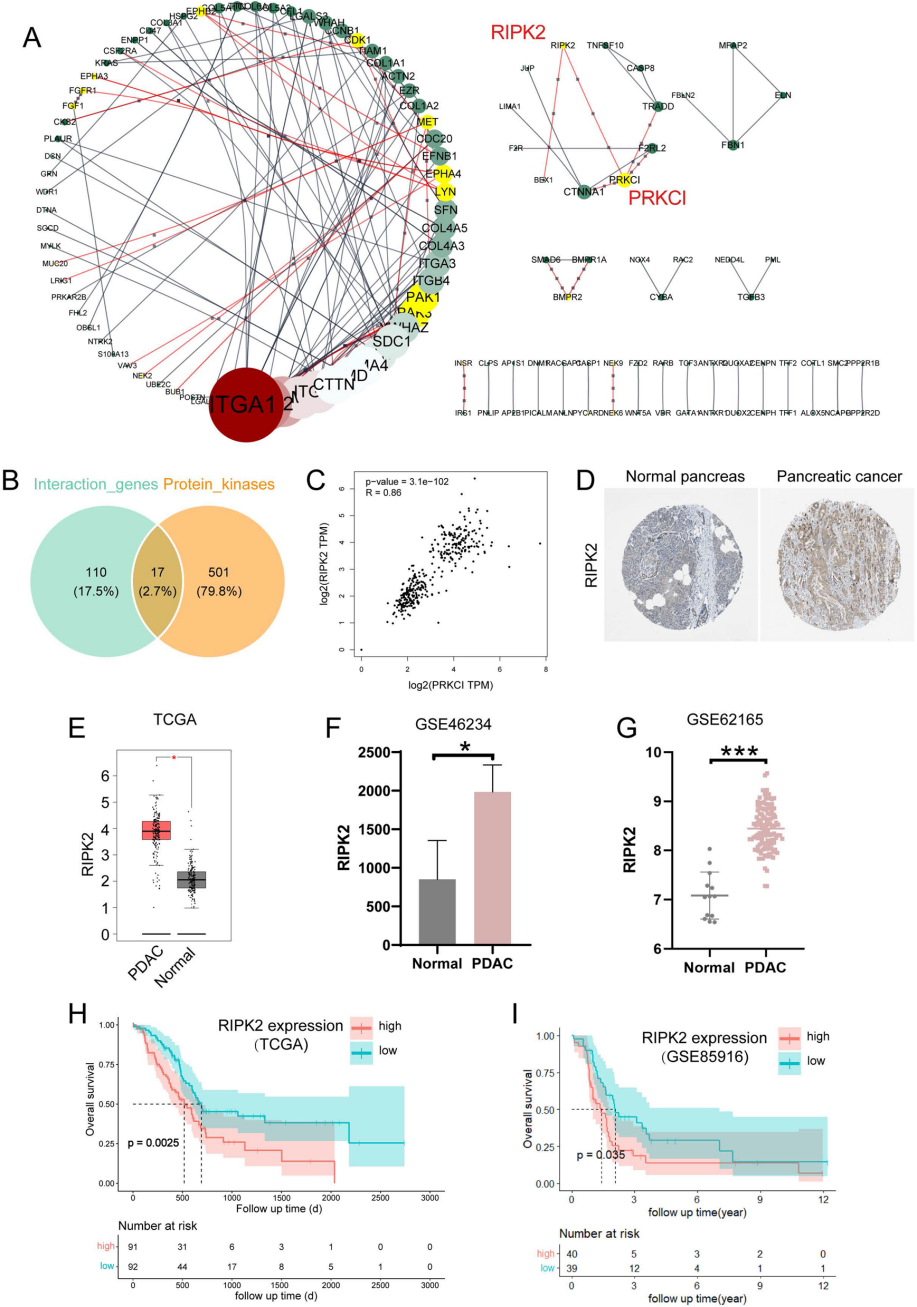
RIPK2 was screened out as a key protein kinase in pancreatic cancer. **A** protein–protein interaction analysis with physical connection (p < 1.0e−16, the highest confidence of 0.9) based on the DEGs. The kinase-encoding genes were marked in yellow and their links in red. **B** Venn diagram indicating the differentially expressed protein kinases. **C** Spearman correlation analysis of the RIPK2 and PRKCI expression in both normal pancreas and pancreatic cancer through GEPIA database (R = 0.86, p = 3.1e−102). **D** RIPK2 protein expression between pancreatic cancer and normal pancreas by immunohistochemistry in the Human Protein Atlas. **E**–**G** RIPK2 mRNA expression between pancreatic cancer and normal tissue based on TCGA and GEO databases. **H**, **I** Survival analysis of RIPK2 in pancreatic cancer patients based on TCGA and GEO databases. ^*^p < 0.05, ^***^p < 0.001

Hub genes were determined by the PPI analysis, and 127 genes were shown to interact with one or more genes in the network (p < 1.0e−16, the highest confidence of 0.9, Fig. 2A). Among these interactive genes, seventeen protein kinase-encoding genes were determined through referring to all of the protein kinases currently identified (Fig. 2B). In these 17 encoded protein kinases, seven kinases belong to the tyrosine kinase family, nine kinases belong to the serine/threonine kinase family, and one kinase that is RIPK2 has both tyrosine and serine/threonine activities (Table 1). Gene expression checked in GEPIA database suggested that 13 protein kinase-encoding genes including BMPR2, BUB1, CDK1, EPHA3, EPHA4, EPHB2, MET, NEK6, PAK1, PRKCI, RIPK2, NEK2, LYN were upregulated, while PAK3 was downregulated in PC than normal tissues. The expression of RIPK2 was highly correlated with the expression of its interacted protein PRKCI (R = 0.86, p = 3.1e−102, Fig. 2C). Overall survival analyses show that the expression of BUB1, CDK1, MET, PAK1, PRKCI, RIPK2, NEK2, FGFR1, NEK9 were significantly correlated with the prognosis of PC patients (Table 1). Considering the multiple kinase activity of RIPK2, and that its high expression in PC predicts a poor prognosis in TCGA database, we next corroborated the expression of RIPK2 at the protein level by immunohistochemistry in the Human protein atlas database (Fig. 2D), and at the mRNA level in independent datasets GSE46234 and GSE62165 (Fig. 2E–G). In addition, the survival significance of RIPK2 was further verified by different datasets from TCGA and GSE85916 (Fig. 2H, I).

**Table 1:** Seventeen differentially expressed protein kinases in pancreatic cancer

No	Gene symbol	Gene name	Type	Expression^a^ (T vs. N)	OS^b^ (p value)
1	BMPR2	Bone morphogenetic protein receptor type 2	Serine/threonine kinase	High	0.58
2	BUB1	BUB1 mitotic checkpoint serine/threonine kinase	Serine/threonine kinase	High	0.0033
3	CDK1	Cyclin dependent kinase 1	Serine/threonine kinase	High	0.0006
4	EPHA3	EPH receptor A3	Tyrosine kinase	High	0.66
5	EPHA4	EPH receptor A4	Tyrosine kinase	High	0.52
6	EPHB2	EPH receptor B2	Tyrosine kinase	High	0.3
7	INSR	Insulin receptor	Tyrosine kinase	NS	0.57
8	MET	MET proto-oncogene	Tyrosine kinase	High	0.00023
9	NEK6	NIMA related kinase 6	Serine/threonine kinase	High	0.29
10	PAK1	P21(RAC1) activated kinase 1	Serine/threonine kinase	High	0.048
11	PAK3	P21(RAC1) activated kinase 3	Serine/threonine kinase	Low	0.25
12	PRKCI	Protein kinase C iota	Serine/threonine kinase	High	0.00063
13	RIPK2	Receptor interacting Serine/threonine kinase 2	Serine/threonine/tyrosine kinase	High	0.0025
14	NEK2	NIMA related kinase 2	Serine-threonine kinase	High	0.0038
15	LYN	LYN proto-oncogene	Tyrosine kinase	High	0.15
16	FGFR1	Fibroblast growth factor receptor 1	Tyrosine kinase	NS	0.028
17	NEK9	NIMA related kinase 9	Serine/threonine kinase	NS	0.049

^a^Gene expression comparison between Tumor and the normal (T vs. N); ^b^ overall survival analysis based on TCGA database

**Fig. 3:**
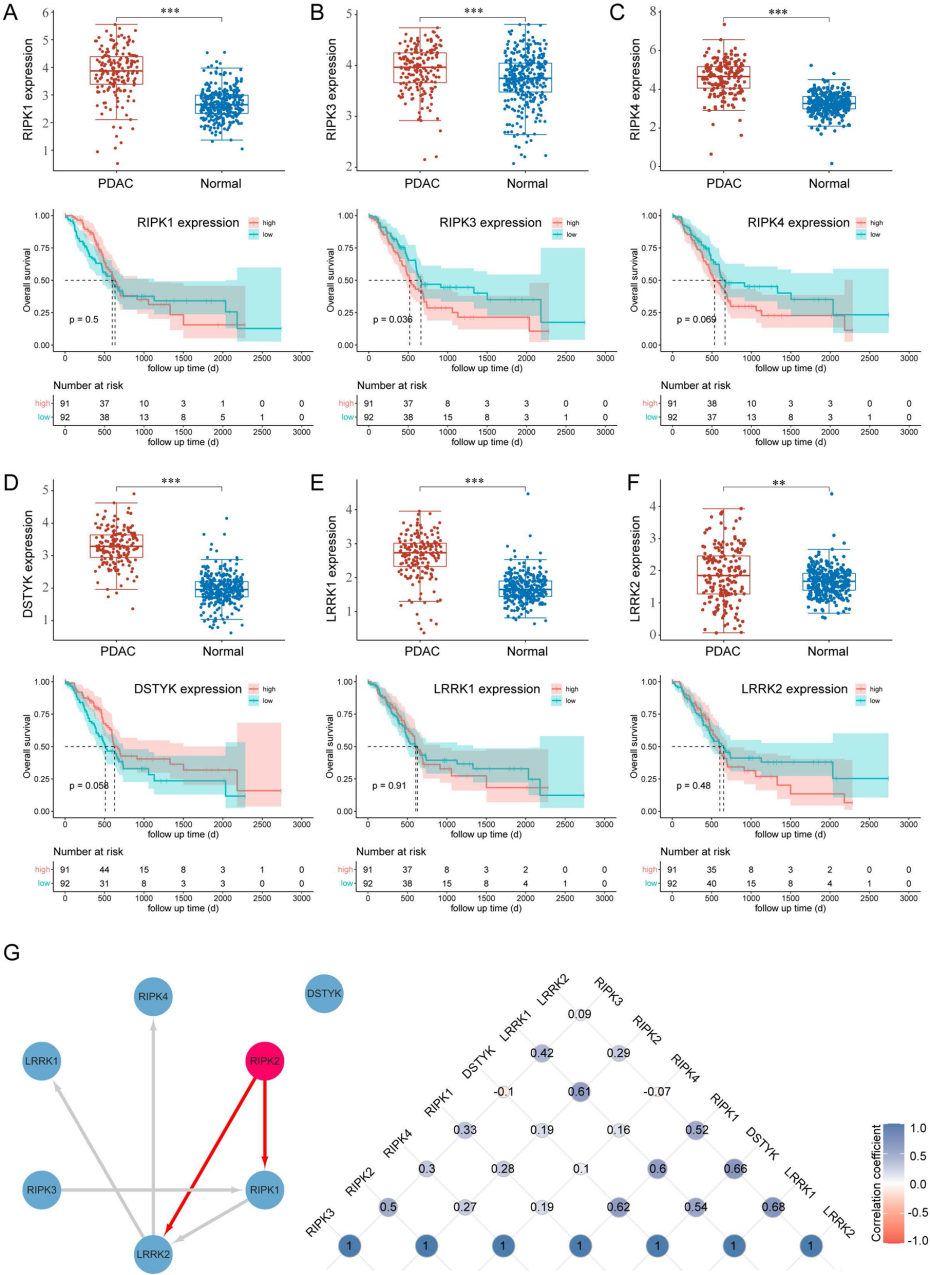
Expression, survival, interacting and correlation analyses of the RIPKs family members. **A** RIPK1 expression between pancreatic cancer and normal tissues and its relevance to the survival of pancreatic cancer patients. **B** RIPK3 expression between pancreatic cancer and normal tissues and its relevance to the survival of pancreatic cancer patients. **C** RIPK4 expression between pancreatic cancer and normal tissues and its relevance to the survival of pancreatic cancer patients. **D** RIPK5 (DSTYK) expression between pancreatic cancer and normal tissues and its relevance to the survival of pancreatic cancer patients. **E** RIPK6 (LRRK1) expression between pancreatic cancer and normal tissues and its relevance to the survival of pancreatic cancer patients. **F** RIPK7 (LRRK2) expression between pancreatic cancer and normal tissues and its relevance to the survival of pancreatic cancer patients. **G** The interactions and correlations among seven members of RIPKs family. **p < 0.01, ***p < 0.001

RIPK2 is a member of receptor-interacting protein kinases (RIPKs or RIPs) family, which were surmised to play a broad range of activities in PC development. RIPK1, RIPK3, RIPK4, DSTYK (RIPK5), LRRK1 (RIPK6), and LRRK2 (RIPK7) are all upregulated in PC than normal tissues (Fig. 3A–F). Survival analyses indicate that the expression of RIPK3 is significantly related to the OS of PC patients (Fig. 3B). Gene interaction and correlation analyses show different degrees of correlation among the members of RIPKs family, and that RIPK2 was predicted to directly target RIPK1 and LRRK2 (Fig. 3G). These results suggested that the members of RIPKs family were aberrantly expressed in PC and there were interactions among them, which may play important roles in PC development and progression.

### RIPK2 overexpression promotes PC cell proliferation, migration and invasion

**Fig. 4:**
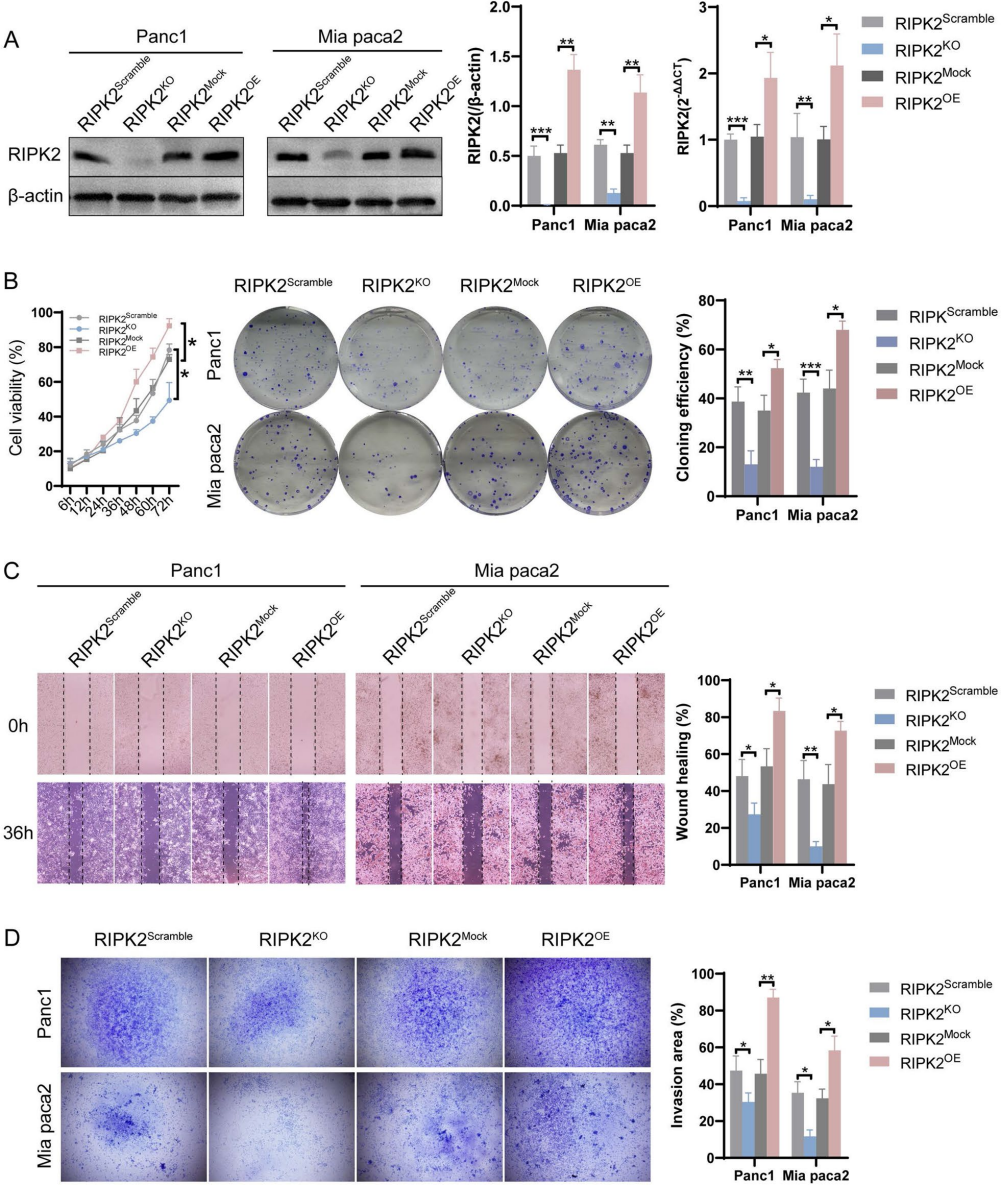
The expression of RIPK2 affected pancreatic cancer cells proliferation, migration and invasion. **A** Validation of the stably transfected RIPK2 knockout cells (RIPK2^KO^) and overexpression cells (RIPK2^OE^). **B** Cell proliferation and colony formation assays of cells. **C** Wound-healing assay of cells. **D** Transwell invasion assay of cells. ^*^p < 0.05, ^**^p < 0.01, ^***^p < 0.001

RIPK2 was stably regulated by a lentiviral transfection method to investigate whether its expression could affect the malignant phenotypes of PC cells. As shown in Fig. 4A, RIPK2 knockout and overexpression were successfully established with Panc1 and Mia paca2 cell lines. Then, cell proliferation and colony formation tests were performed to evaluate cell growth activity. Cells in the RIPK2^OE^ group had an increased cell proliferation rate than cells in the RIPK2^Mock^ group, while cells in the RIPK2^KO^ group had a decreased proliferation rate than cells in the RIPK2^Scramble^ group. Colony formation assay showed there were more cell colonies with more cell numbers in the RIPK2^OE^ group than the RIPK2^Mock^ group, while it was less in the RIPK2^KO^ group than the RIPK2^Scramble^ group (Fig. 4B). Wound-healing assay showed cells in the RIPK2^OE^ group had a higher migration rate than that in the RIPK2^Mock^ group, while cells in the RIPK2^KO^ group had a lower migration rate that that in the RIPK2^Scramble^ group (Fig. 4C). Transwell invasion assay showed more cells in the RIPK2^OE^ group than the RIPK2^Mock^ group to penetrate the compartment, while cells in the RIPK2^KO^ group showed less penetrating cells than cells in the RIPK2^Scramble^ group (Fig. 4D). These results suggested that RIPK2 overexpression promotes PC cell proliferation, migration and invasion, while its knockout inhibited these malignant behaviors.

### The expression of RIPK2 affects PC cell apoptosis and ROS production

**Fig. 5:**
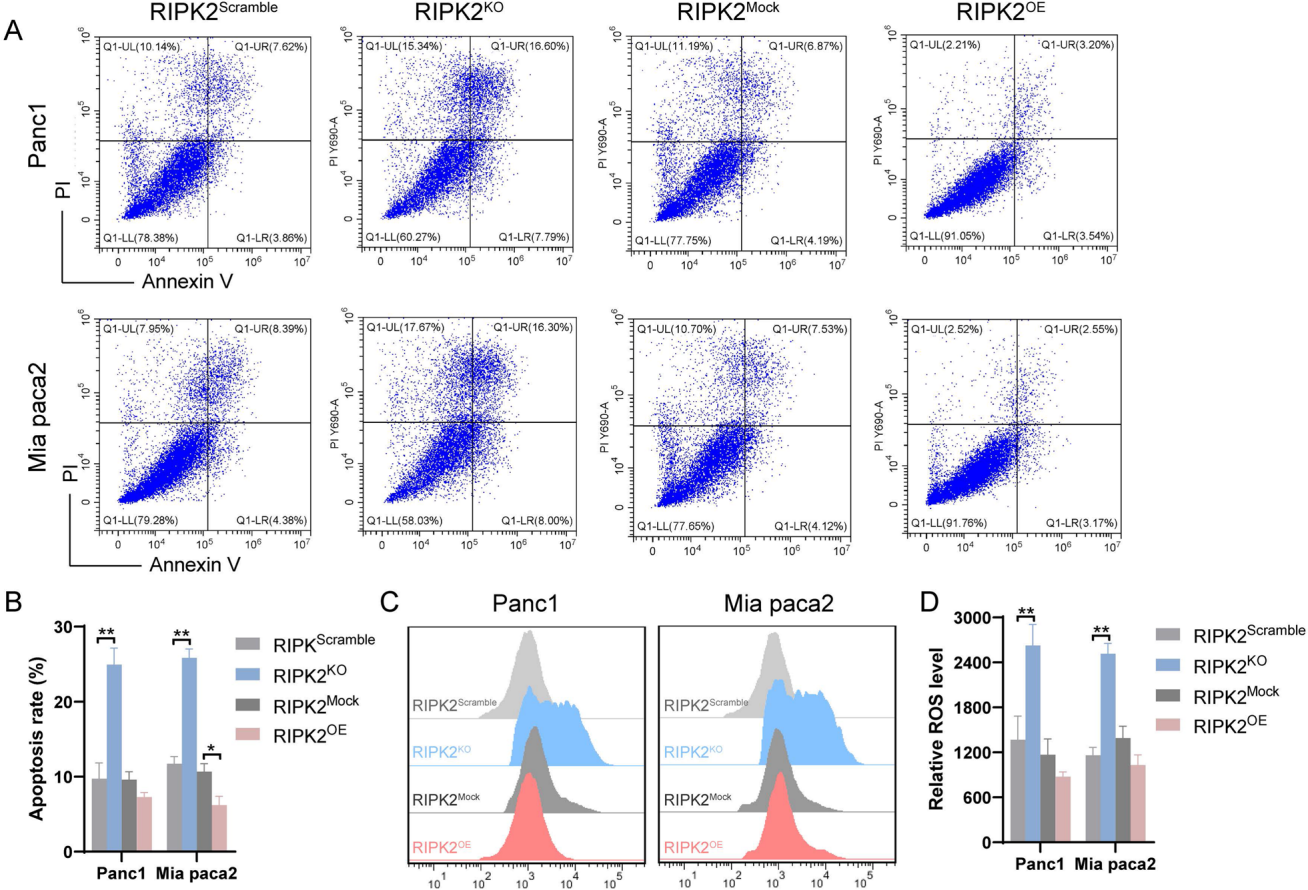
Apoptosis and reactive oxygen species (ROS) level of pancreatic cancer cells. **A** Apoptosis assay of pancreatic cancer cells after RIPK2 knockout and overexpression. **B** Statistical analyses of apoptosis rate of pancreatic cancer cells. **C** ROS level assay of pancreatic cancer cells after RIPK2 knockout and overexpression. **D** Statistical analyses of relative ROS level of pancreatic cancer cells. *p < 0.05, **p < 0.01

Compared with cells in the RIPK2^Scramble^ group, cells in the RIPK2^KO^ group had a higher apoptosis rate. Compared with cells in the RIPK2^Mock^ group, cells in the RIPK2^OE^ showed decreased apoptosis rate for Mia paca2 but not Panc1 (Fig. 5A, B). The relative ROS level of cells in the RIPK2^KO^ group was higher than cells in the RIPK2^Scramble^ group, and no significant difference was observed between the relative ROS level of cells in the RIPK2^OE^ group and cells in the RIPK2^Mock^ group (Fig. 5C, D).

### RIPK2 knockout suppresses pancreatic tumor growth and liver metastasis in vivo

**Fig. 6:**
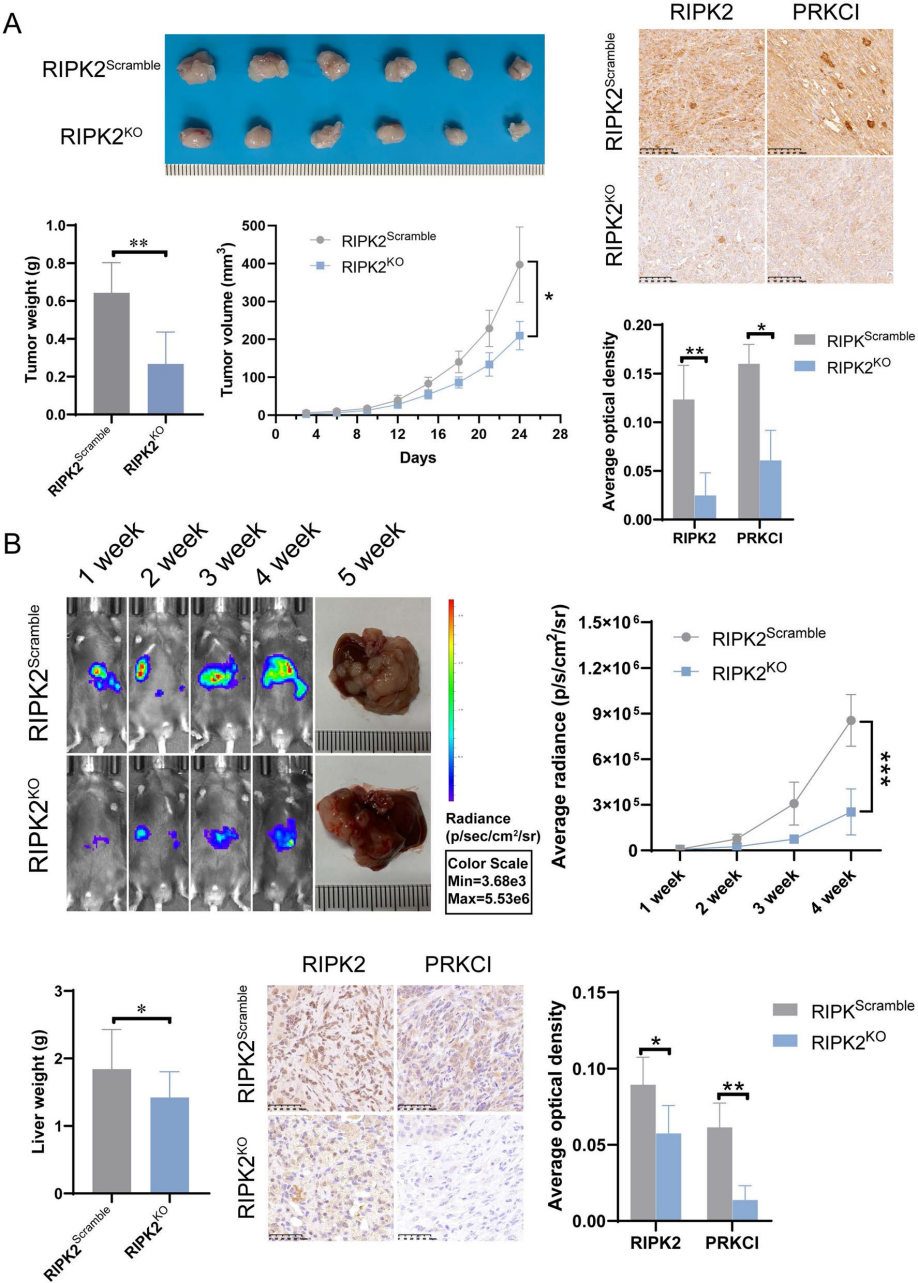
RIPK2 knockout inhibited subcutaneous tumor growth and liver metastasis of pancreatic cancer. **A** Comparison of subcutaneous tumors weight, growth rate, RIPK2 and PRKCI expression. **B** Comparison of liver metastatic rate, and the expression of RIPK2 and PRKCI in the metastatic sites. ^*^p < 0.05, ^**^p < 0.01, ^***^p < 0.001

Observing the apparent influence of RIPK2 expression on the malignant phenotypes in vitro, the in vivo studies were then performed to test whether RIPK2 knockout could inhibit tumor growth and liver metastasis. Compared with mice in the RIPK2^Scramble^ group, mice in the RIPK2^KO^ group showed smaller tumor volume, lower weight and slower tumor growth rate. Both the expression of RIPK2 and PRKCI in tumor tissues decreased in mice of the RIPK2^KO^ group than mice of the RIPK2^Scramble^ group (Fig. 6A). In the liver metastasis mouse model, we observed slower metastasis rate in mice of the RIPK2^KO^ group than mice of the RIPK2^Scramble^ group, and the mouse liver weights in the endpoint of experiment were lower in mice of the RIPK2^KO^ group than mice of the RIPK2^Scramble^ group, with less metastatic foci in mice of the RIPK2^KO^ group than mice of the RIPK2^Scramble^ group. Both the expression of RIPK2 and PRKCI in the metastatic sites decreased in mice of the RIPK2^KO^ group than mice of the RIPK2^Scramble^ group (Fig. 6B). These results suggested that RIPK2 knockout had suppressive roles on the growth and metastasis of PC, in addition, RIPK2 knockout downregulated the expression of RPKCI in both subcutaneous tumors and liver metastatic sites.

### RIPK2 affects other members of RIPKs family and participates in autophagy process

**Fig. 7:**
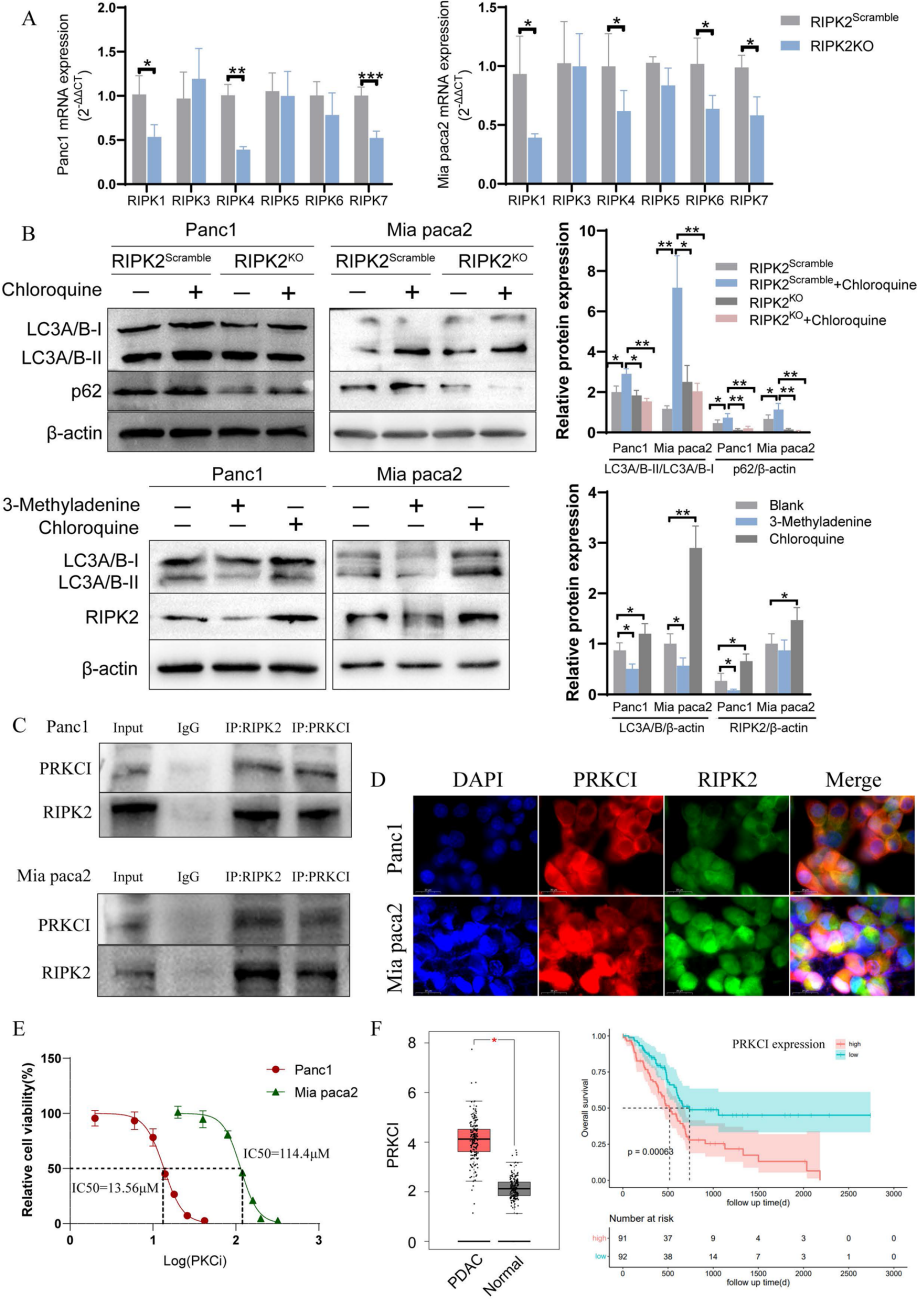
RIPK2 can interact with RIPKs family members and PRKCI, and participate in autophagy. **A** mRNA expression of RIPKs family members. **B** The effects of RIPK2 expression on autophagy, and autophagy on the expression of RIPK2. **C** Coimmunoprecipitation of RIPK2 and PRKCI. **D** Colocalization assay of RIPK2 and PRKCI by immunofluorescence. **E** PKC iota inhibition (PKCi) can decrease pancreatic cell viability. **F** Expression and survival analyses of PRKCI in pancreatic cancer through GEPIA database. ^*^p < 0.05, ^**^p < 0.01, ^***^p < 0.001

After understanding the tumor-promoting role of RIPK2 in PC, we next explored the potential action mechanisms involved. First, the expression of other members of RIPKs in the RIPK2^KO^ cells were analyzed, results showed that RIPK2 knockout inhibited the expression of RIPK1, RIPK4 and RIPK7 (LRRK2) (Fig. 7A). In addition, using the chloroquine to inhibit autophagy, cells in the RIPK2^KO^ group exhibited decreased ratio of LC3A/B-II to LC3A/B-I. Chloroquine increased the expression of p62 in the RIPK2^Scramble^ cells, which was reversed  in the RIPK2^KO^ cells (Fig. 7B). These results suggested that RIPK2 knockout inhibited the formation of autophagosomes. Autophagy inhibitor 3-methyladenine decreased the expression of total LC3A/B and RIPK2, while chloroquine increased the expression total LC3A/B and RIPK2 (Fig. 7B). These results suggested that RIPK2 can regulate the other members of RIPKs family, and mainly participate in the autophagosome formation stage of autophagy in PC.

### RIPK2 can interact with PRKCI in PC

**Fig. 8:**
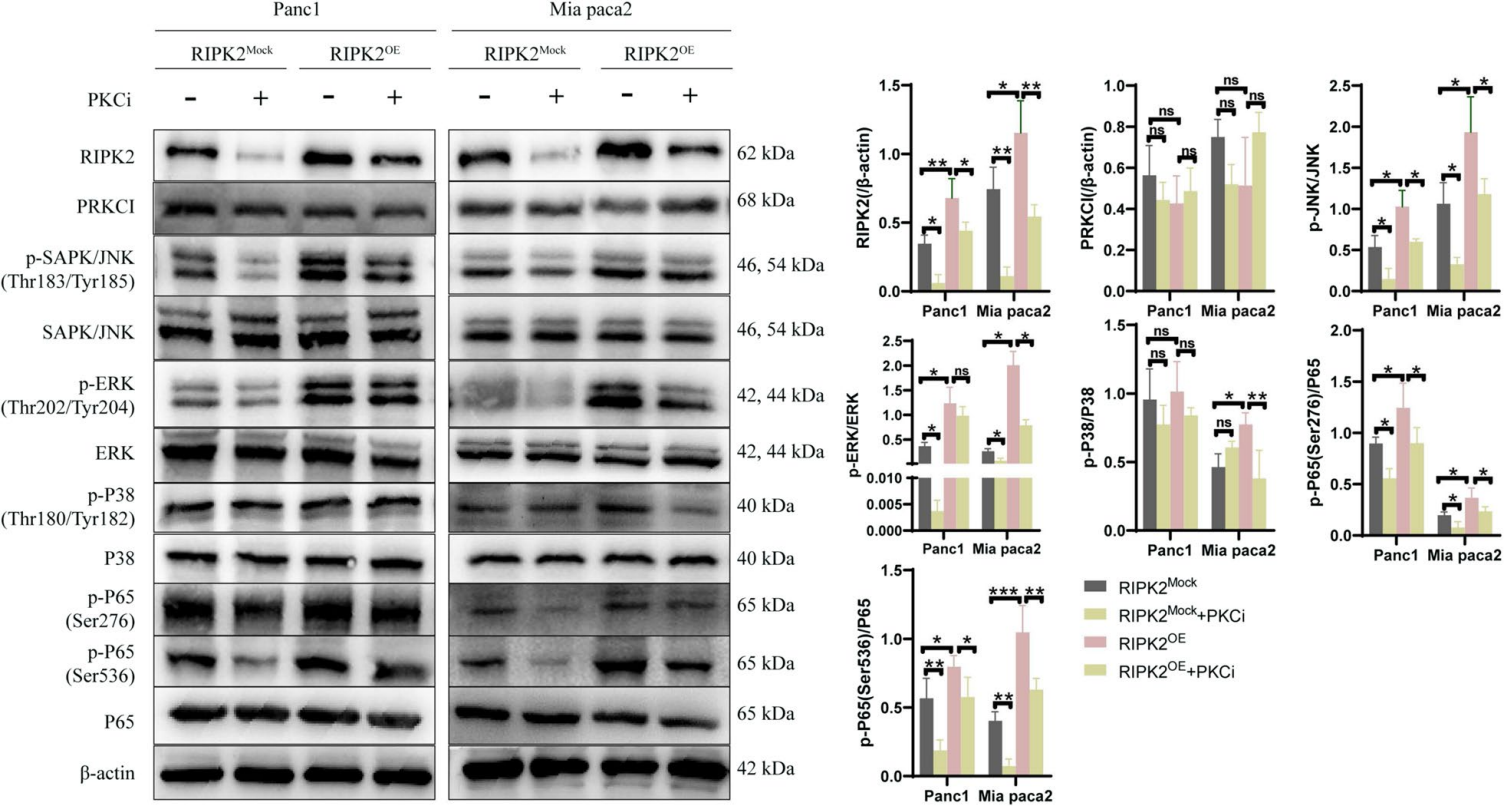
PKC iota inhibition affected the expression of RIPK2 and the phosphorylation of NF-κB, JNK and ERK. ^*^p < 0.05, ^**^p < 0.01, ^***^p < 0.001, ^ns^ not significant

As the PPI analysis with confidence set to 0.9 (Fig. 2A), the physical connection between RIPK2 and PRKCI was highly credible. Besides, the expression between RIPK2 and PRKCI were highly correlated (R = 0.86, p = 3.1e−102, Fig. 2C), and the PRKCI serves as a hub gene based on MCODE modular analysis. We next conducted co-immunoprecipitation to validate the interaction of RIPK2 and PRKCI. As shown in Fig. 7C, protein RPKCI was detected with RIPK2 as the bait protein and vice versa. Meanwhile, immunofluorescence showed colocalization of RIPK2 and PRKCI in PC cells (Fig. 7D). The detailed regulatory pattern was further explored by immunoblotting analysis, results showed no significant difference of PRKCI expression between cells in the RIPK2^OE^ and RIPK2^Mock^ groups (Fig. 8). After that, PKC-iota inhibitor 1 (PKCi), a specific inhibitor of protein kinase C-iota (Kwiatkowski et al. 2019), was used to observe the effect of PKC iota inhibition on the expression of RIPK2. Results showed that PKC iota inhibition induced a significant decrease of RIPK2 expression in both Panc1 and Mia paca2 cells (Fig. 8). Furtherly, when the PKCi was used to interfere with RIPK2 overexpression cells, the expression of RIPK2 was partially reversed (Fig. 8). These results suggested that RIPK2 may serve as the downstream molecule of and could be regulated by PRKCI. Moreover, PKCi could inhibit pancreatic cell viability with an IC50 of 13.56 μM and 114.4 μM for Panc1 and Mia paca2 respectively (Fig. 7E). As already mentioned above, the expression of PRKCI was shown higher in PC than normal tissues (p < 0.05), and its high expression affects the overall survival of PC patients (p = 0.00063, Fig. 7F). Therefore, the PRKCI-RIPK2 interaction may function as a crucial molecular basis in the development and progression of PC.

### PRKCI-RIPK2 interaction is responsible for the phosphorylation of p65, JNK and ERK proteins

As classical pathways downstream of RIPK2, NF-κB signaling and the well-known cancer associated MAPK signaling were evaluated to investigate the specific mechanisms responsible for the RIPK2 induced malignant phenotypes in PC (Fig. 8). The phosphorylation of p65, JNK, ERK and p38 were respectively examined in the RIPK2^OE^ cells and the RIPK2^Mock^ cells. Results showed the phosphorylated p65 at both Ser276 and Ser536 sites were increased in the RIPK2^OE^ group of both cell lines, the same were the phosphorylated JNK (Thr183/Tyr185) and ERK (Thr202/Tyr204). However, the phosphorylated p38 (Thr180/Tyr182) was significantly increased in the RIPK2^OE^ group of only Mia paca2 cells. As PRKCI was suggested as the upstream molecule to regulate RIPK2, the PKCi was used to explore effects of PKC iota inhibition on p65, JNK, ERK and p38. Results showed that PKCi significantly decreased the phosphorylation levels of p65, JNK and ERK, while RIPK2 overexpression reversed these phenomena. Similar trend was observed with the phosphorylated p38 in Panc1 cells, but it was not statistically significant. The inhibition of PKCi on the phosphorylated p38 in the RIPK2^OE^ group of Mia paca2 might be attributed to potential systematic error. These results suggested that RIPK2 partially mediated the inhibition of PKCi on the phosphorylation of p65, JNK and ERK. In other words, PRKCI-RIPK2 interaction could modulate the activity of NF-κB, JNK and ERK signaling.

## Discussion

This study based on independent clinical datasets provides credible data about protein kinases dysregulation in PC. Protein kinases respond to receptor recognition and transfer clues to its downstream messengers, by which to regulate extracellular matrix composition and reconstitution, and cell motility. Stromal desmoplasia represents a typical feature of PC to thwart treatment. Therapeutic regimens targeting stroma have stepped into a dilemma about whether the matrix component should be completely removed (Polani et al. 2021). Restoring and maintaining a homeostatic stromal constitution is a compromise proposal at present. Besides, protein kinases-mediated uncontrolled autonomous movement lay the foundation for invasion and metastasis of PC cells. Therefore, maintaining physiological expression of protein kinases and stabilizing kinase activity can help to shape a favorable stroma and take control of cell motility.

Most of the identified differentially expressed protein kinase-encoding genes are associated with the prognosis of PC patients. In fact, protein kinase-targeting drug development has received much attention despite the unsatisfactory effect achieved in PC treatment to date (Creeden et al. 2020). It is necessary to sort out the intricate mechanisms of protein kinases in PC for treatment improvement. RIPKs family includes seven members with serine/threonine activity and closely participate in the initiation and progression of digestive malignancies, among which RIPK1, RIPK3 and RIPK4 have been studied on their promotive role in PC oncogenesis, apoptosis and necroptosis (Zhang et al. 2021a). RIPK2 is different from other members of RIPKs in its additional tyrosine kinase activity, and currently rarely studied in PC, with only two reports about its prognostic value as autophagy associated gene (Li et al. 2020; Zhang et al. 2021b). Despite limited evidence, pan-cancer analyses highlighted the role of RIPK2 in mediating malignant progression and immunotherapy resistance in multiple tumors (Song et al. 2022; Zhang et al. 2022). As reported so far, RIPK2 could activate NF-κB and JNK signaling to promote cell migration and invasion of triple-negative breast cancer (Singel et al. 2014). Inhibition of RIPK2 can prevent the production of tumorigenic IL-17 in colorectal cancer (Garo et al. 2021). Recent study showed that RIPK2 reduced prostate cancer metastasis through regulating c-Myc stability and activity (Yan et al. 2022). Our study supplemented understanding on the role of RIPK2 in PC, indicating that the expression of RIPK2 was aberrantly increased in PC and a higher expression of RIPK2 predicted a poorer prognosis of PC patients.

The expression of RIPK2 affects the capability of PC cell proliferation, colony formation, migration and invasion. Deletion of RIPK2 inhibits tumor growth and limits the formation of liver metastatic foci. These results demonstrate that RIPK2 functions as a crucial pro-tumor gene and can become a potential intervention target in PC treatment. In addition, the dysregulated RIPK2 expression in PC affected the expression of other members of RIPKs, especially the RIPK1, which was also predicted to be regulated by RIPK3. RIPK1 and RIPK3 can induce necroptosis to drive pancreatic cancer progression (Seifert et al. 2016). Thus, targeting the core kinase in RIPKs family may bring a global regulation role in PC evolution. On the other hand, RIPK2 can activate the autophagy and suppress reactive oxygen species (ROS) production (Gao et al. 2019; Lupfer et al. 2014), which was intimately linked to the kinase activity of RIPK2 itself (Lupfer et al. 2013). RIPK2 self-assembled endosome or RIPosome provide a signal of being eaten in the process of autophagy (Irving et al. 2014; Mehto et al. 2022), this may serve as a scavenger to eliminate ROS in cells. We and other groups have previously shown that increased ROS production can trigger pancreatic cancer cell apoptosis (Lee et al. 2022; Wang et al. 2021). Here, we verified that RIPK2 knockout can suppress the formation of autophagosomes, elevate ROS level and promote PC cell apoptosis.

Furtherly, PRKCI was found to interact with RIPK2 in PC. Similar to RIPK2, PRKCI also has a higher expression in PC tissues than the normal, and a higher PRKCI expression predicts a poorer survival of PC patients. The oncogenic role of PRKCI-encoding protein PKC iota has been well established, recent studies in PC suggested that mutated KRAS can elevate and activate PKC iota to disable growth-inhibitory Hippo signaling and promote Yes-associated protein1 (YAP-1) translocation into nucleus, and thus maintain PC cells growth (Wang et al. 2020). Also, PKC iota upregulates transcription factor specificity protein 1 to promote transactivation of YAP-1 and induce tumorigenesis (Yang et al. 2021). In turn, the produced YAP-1 can be recruited by PKC iota to increase PD-L1 expression, resulting in immune evasion under the microenvironment of PC (Zhang et al. 2021c). These studies suggest a YAP-1 dependent manner in PKC iota driven carcinogenesis, growth and immune tolerance of PC. Here, we found RIPK2 as an interacting kinase with PKC iota, this paired protein kinases PRKCI-RIPK2 represent a novel mechanism to promote malignant behaviors of PC.

RIPK2 has both serine/threonine kinase and tyrosine kinase activities. Its downstream molecule NF-κB is often phosphorylated at serine sites and MAPKs at threonine and tyrosine sites to perform transcription regulation in cancer (Dhillon et al. 2007; Motolani et al. 2020). Our further studies revealed that NF-κB, JNK and ERK signaling contribute to RIPK2 mediated cell growth, migration and invasion in PC. NF-κB pathway has been suggested to increase PC cell motility and epithelial-mesenchymal transition to promote metastasis (Cui et al. 2021).Compounds inhibiting NF-κB signaling could attenuate PC (Cykowiak et al. 2021). JNK signaling is entwined with carcinogenesis and progression in many cancers including PC, making JNK-targeting strategies very promising in cancer management (Wu et al. 2020). The importance of ERK cannot be overemphasized since it is a member of the three-tiered RAF-MEK-ERK cascade under KRAS signaling whose high mutation frequency is the biggest perpetrator of PC (Diehl et al. 2022). Here, Ser576/Ser276 of NF-κB p65, Thr183/Tyr185 of JNK and Thr202/Tyr204 of ERK were validated to be the enhanced phosphorylation sites by RIPK2 overexpression, which were partially reversed by PKC iota inhibition. Thus, PRKCI-RIPK2 interaction at least in part enhances NF-κB, JNK and ERK signaling via their serine/threonine kinase and tyrosine kinase activities to promote PC cell growth, migration and invasion. Other undetermined sites potentially contributing to the activation of NF-κB and MAPKs signaling warrant further investigations.

A limitation in our study lies in that the detailed interacting mode between protein kinases RIPK2 and PKC iota was not clarified. This study focuses on the expression level of protein kinases, without figuring out the mutual influences of enzymatic activity between RIPK2 and PRKCI. Moreover, RIPK2 can auto-phosphorylate itself to become activated (Ellwanger et al. 2019), so the significance of enzymatic activity of this paired protein kinases may outweigh their expression level, which were being investigated in our ongoing work. In addition, there seems to be a contradiction between the in vivo experiment that RIPK2 knockout decreased the expression of PRKCI and the in vitro experiment that RIPK2 overexpression did not affect the expression of PRKCI, this imply that the complicated tumor microenvironment in vivo affects the regulation of protein kinases. The special tumor microenvironment of PC is one of the key factors for its refractory feature and drug resistance, so the PC inhibition by RIPK2 mediated tumor microenvironment remodeling is intriguing to be explored.

## Conclusion

In summary, this study reveals key protein kinases involved in the malignant behaviors of PC. Among them, RIPK2 with multiple enzymatic activities has a promotive role in PC cell proliferation, migration, invasion and metastasis. Another key oncogenic protein kinase PKC iota was found to interact with RIPK2 and enhance the phosphorylation of NF-κB, JNK and ERK. The multiple phosphorylation activities of this paired PRKCI-RIPK2 kinases link their high expression to a poor prognosis of PC patients, which can provide insights into drug research and development related to PC therapy.

## Data Availability

The datasets analysed during the current study are available in the the GEO database (https://www.ncbi.nlm.nih.gov/geo). Pancreatic patients’ information in the TCGA database can be found in the PAAD dataset through GEPIA (http://gepia.cancer-pku.cn/index.html).

## Abbreviations

PC: Pancreatic cancer
EGFR: Epidermal growth factor receptor
PKC: Protein kinase C
PI3K: Phosphoinositide 3 kinase
MAPK: Mitogen-activated protein kinase
DEG: Differentially expressed gene
GO: Gene Ontology
KEGG: Kyoto Encyclopedia of Genes and Genomes
GEO: Gene Expression Omnibus
TCGA: The Cancer Genome Atlas
GEPIA: Gene Expression and Profiling Interactive Analysis
PPI: Protein–protein interaction
RIPK: Receptor interacting protein kinase
ROS: Reactive oxygen species
YAP-1: Yes-associated protein-1
